# Quantifying Unknown Multiqubit Entanglement Using Machine Learning

**DOI:** 10.3390/e27020185

**Published:** 2025-02-12

**Authors:** Yukun Wang, Shaoxuan Wang, Jincheng Xing, Yuxuan Du, Xingyao Wu

**Affiliations:** 1 Beijing Key Laboratory of Petroleum Data Mining, China University of Petroleum, Beijing 102249, China; 2 JD Explore Academy, Beijing 101111, China

**Keywords:** quantifying unknown entanglement, multiqubit states, squared entanglement, machine learning, local measurement

## Abstract

Entanglement plays a pivotal role in numerous quantum applications, and as technology progresses, entanglement systems continue to expand. However, quantifying entanglement is a complex problem, particularly for multipartite quantum states. The currently available entanglement measures suffer from high computational complexity, and for unknown multipartite entangled states, complete information about the quantum state is often necessary, further complicating calculations. In this paper, we train neural networks to quantify unknown multipartite entanglement using input features based on squared entanglement (SE) and outcome statistics data produced by locally measuring target quantum states. By leveraging machine learning techniques to handle non-linear relations between outcome statistics and entanglement measurement SE, we achieve high-precision quantification of unknown multipartite entanglement states with a linear number of measurements, avoiding the need for global measurements and quantum state tomography. The proposed method exhibits robustness against noise and extends its applicability to pure and mixed states, effectively scaling to large-scale multipartite entanglement systems. The results of the experiment show that the predicted entanglement measures are very close to the actual values, which confirms the effectiveness of the proposed method.

## 1. Introduction

The rapid advancement of quantum information has brought considerable attention to quantum entanglement [1], which serves as the cornerstone technology in this field. Many theoretical foundations of quantum information technology, including quantum key distribution [2], quantum teleportation [3], quantum simulation [4], and quantum true randomness [5], are highly dependent on quantum entanglement. Additionally, quantum entanglement is key to comprehending many peculiar properties of quantum mechanics, such as quantum non-locality and quantum many-body systems. Given the crucial role of entanglement, it is important to explore entanglement theory and experimental methods for generating entangled states.

So far, enormous progress has been made in the generation of entanglement. For example, 219 beryllium ions [6], 14-photon entanglement [7], 500-cold-atom entanglement [8], and 51-qubit superconducting entanglement [9] have been generated. With the continuous improvement in the fundamental technology of quantum control, it is expected that larger quantum systems will soon become entangled. However, a common concern in experiments is ensuring that the underlying entanglement is indeed produced, especially for larger systems. This results in the quantum states produced by the experiment being unknown to us and requiring further verification or quantification. Confirming the presence or quantifying the degree of entanglement presents challenges because they cannot be directly observed by any physical device. Various approaches can be considered to address this problem [10,11,12,13,14,15,16,17,18,19,20,21,22,23,24,25]. However, some methods [11,12,13,14,15,16,17] can only detect entanglement without quantification, while others [18,19,20,21,22,23,24,25], although capable of entanglement quantification, face limitations due to the high computational complexity. Quantum state tomography (QST) [26,27,28,29] is a standard method to reconstruct a quantum state by observing its density matrix. Then, based on the density matrix, the existence (with the entanglement criterion [16]) or the quantification of the entanglement (with the entanglement measures [18,19,20,21,22,23,24,25]) can be calculated for the states generated in the experiments. However, such a procedure requires a large number of measurements that scale exponentially with the dimension of the quantum state. Although researchers have proposed compressed sensing methods [28,30] to improve the efficiency of QST by reducing measurement resources, the measurement settings for QST still require exponential resources.

In view of this, one may imagine a new scheme with desirable properties. First, a crucial requirement is that the scheme under consideration must be easy to implement and most importantly allow for fewer and easier-to-implement measurements. In addition, the scheme should be robust to noise. Moreover, most experiments today aim to generate an entanglement between more than two particles. Therefore, the scheme has to be capable of multipartite scenarios. Classical machine learning methods have been proven to be a good scheme for detecting and quantifying entanglements. The idea is to train the neural network with the outcome statistics data produced by measuring sample quantum states as features and the entanglement criterion or measures as sample labels. Ma [31] and Gao [32] used neural networks to optimize the coefficients of the CHSH inequality [15] theoretically and experimentally so that more entanglement states violated the optimized CHSH inequality; however, this method cannot quantify the entanglement; that is, it only answers yes or no. Lin [33,34] successfully employed neural networks to utilize collective measurement probabilities of quantum states as feature inputs, achieving high-precision quantification of coherent information [35] and relative entropy of entanglement [36] in qudit systems. In addition, they applied variational quantum algorithms [37] to seek optimal measurement strategies. However, when Lin [33] attempted to quantify the GME of multipartite systems, the number of measurements they used grew exponentially. Furthermore, due to the complexity of GME computation, this method does not apply to noisy quantum states and is difficult to extend to multipartite systems. Moller [38] quantified the GME of multipartite pure state systems using variational quantum algorithms. By successfully implementing this method on the IBM Quantum systems, they demonstrated its effectiveness in producing GHZ states using three, four, and five qubits. However, challenges remain when dealing with noisy quantum states. Researchers [39,40,41] also quantified different entanglement measures, but their scalability to multipartite and applicability to noisy quantum states are weak.

This paper is based on squashed entanglement to establish a connection between local measurements and entanglement utilizing neural networks. The study accurately quantifies unknown multipartite entanglement states, demonstrating their effectiveness in both pure and noisy quantum states. This method offers remarkable applicability and efficiency by requiring a linear number of measurements, presenting an innovative approach to precisely quantifying unknown multipartite entanglement states. The structure of this paper is as follows: In Section 2, we describe the selection of entanglement measures and measurement settings. The quantification of the entanglement of pure and mixed states is presented in Section 3 and Section 4, respectively. Finally, Section 6 contains conclusions and future work.

## 2. Entanglement Measurements and Measurement Setting

Although extensively studied, methods for entanglement detection typically offer only a relative magnitude of the entanglement and struggle to provide precise numerical values. For instance, CHSH inequality [15] and entanglement witness [17] rely on the degree of violation of inequalities to assess the relative magnitude of entanglement, while the PPT criterion [16] utilizes the minimum eigenvalue for this purpose. However, these methods do not precisely quantify entanglement despite being effective in detection. In recent years, various entanglement measures have emerged, offering flexible tools for comprehensive understanding and characterization of entanglement. However, exact computable measures for multipartite quantum states, particularly mixed states, remain limited due to the exponential growth of the state space and the inherent complexity of capturing the intricate correlations between multiple subsystems. Furthermore, the probabilistic pure decompositions of mixed states and the lack of efficient algorithms further complicate the computation of these measures. This paper focuses on employing neural networks to quantify unknown multipartite entanglement states, despite challenges in preparing training data. To ensure optimal performance, we first reviewed several established entanglement measures suitable for quantifying multipartite states, ultimately opting for squashed entanglement (SE) as the sample label. This decision is driven by SE’s ability to facilitate straightforward calculations for pure states and provide an approximate value for mixed states. Once the label is determined, we provide the data features for the neural network.

### 2.1. Entanglement Measurements

We first review Geometric Measure of Entanglement (GME). Known for its robustness against quantum noise, GME is often more manageable than other measures when dealing with large quantum systems, which has led to its extensive study and application. Initially introduced for bipartite states [18], it was later extended to multipartite scenarios [19]. For a *n*-qubit pure state |ψ〉, GME is defined as follows:

(1) $$E_{p}(|\psi \rangle )=1-{\left(\min_{\phi \in {\mathrm{seq}}_{n}}∥\langle \psi |\phi \rangle ∥\right)}^{2},$$

where ${\mathrm{seq}}_{n}$ is the set of *n*-qubit pure states, and for a mixed state ρ, GME is defined as follows:

(2) $$E_{m}\left(\rho \right)=\min_{\rho =\sum_{i}p_{i}|\psi_{i}\rangle \langle \psi_{i}|}\sum_{i}E_{p}(|\psi_{i}\rangle ).$$

GME measures the distance to separable states, requiring the traversal of the entire set of separable states for computation. Commonly, gradient descent methods and their variants [42,43] are employed for GME computation. However, as *n* grows, challenges in processing optimal programs arise, such as extended computation time and unstable results. Although quantum variational algorithms have been applied to GME calculations [38], the classical parameter optimization process is not inevitable. In addition, when handling mixed states or unknown states, the problem is even more difficult. Therefore, for systems with a larger number of qubits, particularly for larger mixed states, GME often is not the optimal choice.

Measurements that do not require optimization may be more appropriate. Global Entanglement (GE) is one such measurement that avoids optimization and was proposed by Meyer [20]. For an *n*-qubit pure state $|\psi \rangle =\sum_{i=0}^{2^{n}-1}c_{i}|a_{1}\ldots a_{n}\rangle$, $a_{i}\in \left\{0,1\right\}$, GE is defined as follows:

(3) $$Q(|\psi \rangle )=\frac{4}{n}\sum_{i=1}^{n}D\left(\Upsilon_{i}(0,|\psi \rangle ),\Upsilon_{i}(1,|\psi \rangle )\right),$$

Here, $\Upsilon_{i}(b,|a_{1}\ldots a_{n}\rangle )=\delta_{ba_{i}}|a_{1}\ldots a_{i-1}a_{i+1}\ldots a_{n}\rangle ,$ with b∈{0,1}, maps an *n*-qubit quantum state to two *n*-1-qubit quantum states, $u=\sum_{i=0}^{2^{n-1}-1}u_{i}|i\rangle$ and $v=\sum_{j=0}^{2^{n-1}-1}v_{j}|j\rangle$. The distance is defined as $D\left(u,v\right)=\sum_{x<y}{∥u_{x}v_{y}-u_{y}v_{x}∥}^{2}.$ Through Equation (3), it can be seen that GM can be accurately calculated without traversing all the separable states or employing optimization algorithms. However, its computational form is based on the state vector, making it suitable only for pure states. In the case of mixed states, one must use a density matrix, which renders GE unsuitable.

Next, we turn our attention to another multipartite entanglement measurement, the squashed entanglement (SE). Squashed entanglement, also called conditional mutual information (CMI) entanglement, is an information-theoretic measure of quantum entanglement for a bipartite quantum system. It has its roots in classical (non-quantum) condition mutual information theory. Thus, SE was initially introduced to quantify bipartite entanglement [21] and was later extended to its multipartite version [22]. Two variants of SE are described in [22]: q-squashed entanglement and c-squashed entanglement. Although the computational expressions for both q-SE and c-SE are identical for pure states, calculating q-SE for mixed states proves to be more complicated than calculating c-SE. Given this, we choose the c-squashed entanglement as our preferred label.

For *n* party state $\rho_{A_{1},\ldots ,A_{n}}$,

(4) $$E_{sq}^{c}\left(\rho_{A_{1},\ldots ,A_{n}}\right)=\inf\;I\left(A_{1}:A_{2}:\cdots :A_{n}|E\right)$$

where infimum is taken over the extension state $\sigma_{A_{1},\ldots ,A_{n}E}$ of the form $\sum p_{i}\rho_{A_{1},\ldots ,A_{n}}^{i}⊗|i\rangle \;{\langle i|}_{E}$.

The multipartite conditional mutual information has form

(5) $$\begin{array}{cc} & I(A_{1}:A_{2}:\cdots :A_{n}|E)\\ & =I\left(A_{1}:A_{2}|E\right)+I\left(A_{3}:A_{1}A_{2}|E\right)+\cdots +I\left(A_{n}:A_{1}\ldots A_{n-1}\right)\\ \; & =\sum_{i=1}^{n}S\left(A_{i}E\right)-S\left(A_{1}A_{2}\ldots A_{n}E\right)-\left(n-1\right)S\left(E\right) \end{array}$$

where S(A) is the von Neumann entropy, and S(A)=−tr(AlogA). With entropy property calculation, we have

(6) $$\begin{array}{cc} I(A_{1}:A_{2}:\cdots :A_{n}|E) & =\sum_{i}\sum_{j}p_{i}S\left(\rho_{A_{j}}^{i}\right)-\sum_{i}p_{i}S\left(\rho^{i}\right)\\ \; & \geq \sum_{i}\sum_{j}p_{i}S\left(\rho_{A_{j}}^{i}\right)-S\left(\rho \right) \end{array}$$

where we use the joint quantum entropy theorem for each $S(A_{i}E)$ and $S(A_{1}A_{2}\ldots A_{n}E)$

(7) $$S(\sum_{i}p_{i}|i\rangle \langle i|⊗\rho_{A_{1},A_{2}\ldots ,A_{n}}^{i})=H\left(p_{i}\right)+\sum_{i}p_{i}S\left(\rho_{A_{1},A_{2},\ldots ,A_{n}}^{i}\right)$$

and entropy inequality

(8) $$S\left(\sum_{i}p_{i}\rho^{i}\right)\geq \sum_{i}p_{i}S\left(\rho^{i}\right)$$

As we can see, for pure state $\rho_{A_{1}A_{2},\ldots ,A_{n}}$, it has

(9) $$E_{sq}^{c}\left(\rho_{A_{1},\ldots ,A_{n}}\right)=\sum_{j}S\left(\rho_{A_{j}}\right)$$

where S(ρ)=0. Choosing normalization factor $\frac{1}{n}$, we can obtain 1 for the GHZ state. For $\rho_{A_{1}A_{2},\ldots ,A_{n}}^{i}$, when they are pure states, Equation (6) is the case of minimum; otherwise, by denoting $\rho^{i}=\sum_{k}p_{k}^{i}|\psi_{k}^{i}\rangle \;\langle \psi_{k}^{i}|\;\mathrm{and}\;\;\rho^{i,k}=|\psi_{k}^{i}\rangle \;\langle \psi_{k}^{i}|$, we have

(10) $$\begin{array}{cc} E_{sq}^{c} & \geq \sum_{i}p_{i}(\sum_{j}\sum_{k}p_{k}^{i}S\left(\rho_{A_{j}}^{i,k}\right)-S\left(\rho \right)\\ \; & =\min_{\left\{p_{i},\rho_{i},p_{k}^{i},\rho^{i,k}\right\}}\sum_{i,k}p_{i}p_{k}^{i}\sum_{j}S\left(\rho_{A_{j}}^{i,k}\right)-S\left(\rho \right) \end{array}$$

In principle, running over all pure-state decompositions of $\rho =\sum_{i,k}p_{i}p_{k}^{i}|\psi_{k}^{i}\rangle \;\langle \psi_{k}^{i}|$ would give a lower bound of c-SE for the mixed states.

In summary, for quantifying the entanglement of multipartite states, including multipartite mixed states, SE demonstrates its ability to facilitate straightforward calculations for pure states while providing an approximate lower value for mixed states. This makes it a suitable choice for preparing training data. While Lin’s study [33] has shown the possibility of predicting unknown multipartite entanglement based on GME, it primarily focuses on three-qubit systems, and as we have mentioned, extending it to multipartite states is challenging. Furthermore, we should also acknowledge that mutual information [41] has been explored as an entanglement measure to measure entanglement. However, while it is easy to calculate, it can yield a negative value for mixed states, which means it is not a proper entanglement measure. This further justifies our preference for SE.

Although the calculation of Equation (10) only provides a lower bound for SE in mixed states, it still holds practical significance as it indicates the least amount of SE entanglement in the system. Moreover, it is crucial to note that different entanglement measures applied to the same state can yield different values. In some cases, the exact value of entanglement may not fully capture the extent of the entanglement. The entanglement measure itself remains an area of ongoing investigation.

### 2.2. Measurement Setting

From Equation (9), the c-SE for a pure state can be computed by taking the von Neumann entropy of each single subsystem, which can be represented using the reduced density matrix of each qubit. Since local observables provide an effective way to compute the von Neumann entropy of these subsystems, measurements X,Y, and *Z* are therefore efficient in determining the entanglement bound for the pure state. This efficiency arises because S(ρ) is zero for pure states. When dealing with mixed states in quantum systems, determining the value of S(ρ) typically requires information-complete measurements over bipartite quantum systems. This involves each party randomly choosing X,Y and *Z* and measuring simultaneously to obtain global statistics, such as XX,XY,XZ,YX,YY,YZ,ZX,ZY, and ZZ, resulting in a the total number of 9 statistics for the scenario of two parties. These simultaneous measurements are considered special global measurements. In the case of multipartite systems, an exponential number of global measurements would be required.

Here, we select the expected values of local measurements as the features of the neural network. For example, for a three-qubit system, the measurement operators are XII, YII, ZII, IXI, IYI, IZI, IIX, IIY, and IIZ. For an *n*-qubit system, we will have the input neural network with expected values of 3n. Assuming that each party *i*, where i∈{1,…,n}, has a set of measurement devices labeled by $U_{j}$, the measurement operators can be defined as follows:

(11) $$\sigma_{i}={⊗}_{j=1}^{n}\left\{\begin{array}{c} U_{j}\in \left\{X,Y,Z\right\},\;\mathrm{if}\;j=i\\ U_{j}=I,\;\;\;\;\;\mathrm{otherwise} \end{array}\right..$$

We want to assess how well local measurements can quantify the entanglement of an unknown multipartite quantum state using neural networks. The neural networks will learn and extract features of local measurement operations to build a function mapping related to SE. This will help model the potential relationship between the SE and local measurements.

In fact, machine learning methods have already been introduced to solve quantum information problems, such as detecting problems of non-locality [44], steerability [45], fidelity [46], and quantum error mitigation [47] problems. In these studies, researchers have discovered that artificial neural networks can achieve high accuracy with fewer measurement settings, thereby identifying inherent information within the quantum state space. Leveraging the powerful ability of neural networks to handle non-linear relationships and complex data structures, they have been applied to the quantification of entanglement in quantum systems [33,34,40,41]. This work focuses on quantifying unknown entanglement states using neural networks, especially for larger systems of entanglement states that are expected to be observed in experiments. Relevant studies have thoroughly verified the feasibility of this approach, providing a solid foundation for further exploration and application of neural networks in quantifying entanglement states in quantum systems.

Additionally, Lin [33] used collective (or joint) measurements of the following form when predicting GME:

(12) $$p\left(a_{1}a_{2}\ldots a_{n}|x_{1}x_{2}\ldots x_{n}\right)=Tr\left({⊗}_{i=1}^{n}M_{x_{i}}^{a_{i}}\rho \right),$$

where each party randomly chose *M* from $\left\{\sigma_{x},\sigma_{y},\sigma_{z}\right\}$. Thus, there are a total of $3^{n}$ possible combinations of measurements. In contrast to the collective measurements required for $3^{n}$ possible combinations, the advantages of the measurement setting chosen in this paper for SE are evident.

## 3. Quantifying Multipartite Entanglement Pure State Without Noise

**Figure 1:**
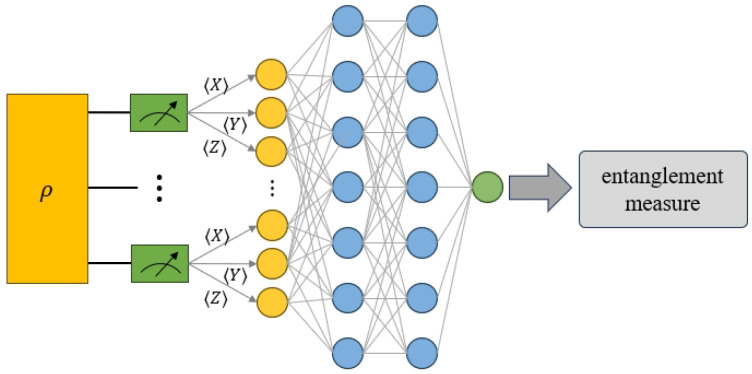
In our work, we employ machine learning to quantify unknown multiqubit entanglement. The process begins with generating random quantum states and collecting observed values from local measurements, which serve as feature inputs for the neural network. We also calculate the c-SE of the target quantum state to use as the network’s label.

In the preceding description, the features and labels of the neural network have been introduced. At this stage, numerical simulation experiments will be conducted in pure states to verify whether the measurement settings in Equation (11) can be used to predict the SE of the unknown multipartite quantum state. The workflow is illustrated in Figure 1. We will employ random quantum states for training the neural network. The true SE values serve as labels, computed with Equation (9), and the local measurement results, specified in Equation (11), provide the features of these training states. Then, we will continuously optimize the parameters of the neural network to train it to predict the SE for unknown entanglement states.

We first generate a series of random state $|\varphi_{e}\rangle$ which has the following form:

(13) $$|\varphi_{e}\rangle =\frac{\sum_{j=0}^{2^{n-1}}c_{j}|j\rangle }{∥|\varphi_{e}\rangle ∥},$$

where the real and imaginary parts of the elements $c_{j}=a_{j}+b_{j}i$ are generated by a uniform distribution in the interval −1 to 1. The formula for calculating the SE of the pure state is Equation (9). Since the SE of most of these states is very close to 1, it shows the entanglement of the randomly generated states is not uniformly distributed in the interval 0 to 1. To ensure that the trained neural network is able to predict all entanglements in 0 to 1, we then generate some random separable state:

(14) $$|\varphi_{s}\rangle =\frac{(c_{0}|0\rangle +c_{1}{\left|1\rangle \right)}^{⊗n}}{∥|\varphi_{s}\rangle ∥},$$

and introduce a parameter t∈[0,1] to make our random quantum state |φ〉 look like the following form:

(15) $$|\varphi \rangle =\frac{t|\varphi_{e}\rangle +\left(1-t\right)|\varphi_{s}\rangle }{∥|\varphi \rangle ∥}.$$

|φ〉 is the random quantum state we used for training the neural networks. For preparing the training samples, when we generate a random quantum state, we measure it and calculate its true entanglement with the SE formula in Equation (9). We set the SE ∈[0,1] with a step size of 0.1, and for each step, we choose 1000 random quantum states with this entanglement, resulting in a total of 10,000 training data.

This section focuses mainly on the performance of the neural network on multipartite systems without noise. We then describe the configuration parameters of the neural network selected in this work. The neural network is a network structure consisting of an input layer, an output layer, and several hidden layers, each containing multiple neurons. In this paper, a fully connected neural network is chosen, consisting of six hidden layers with 100, 200, 400, 500, 200, and 100 neurons, respectively. The input layer has 3n neurons, and the output layer has one neuron. The non-linear activation function, $\sigma_{RL}$, which can be defined as

(16) $$\sigma_{RL}\left(x\right)=\max\left(x,0\right),$$

is used between each layer. The loss function we chose is the Mean Square Error (MSE), which can be defined as follows:

(17) $$MSE=\frac{\sum_{i=1}^{N}{\left(Y_{i}-{\hat{Y}}_{i}\right)}^{2}}{N},$$

where $Y_{i}$ is the exact value, ${\hat{Y}}_{i}$ is the prediction value, and *N* is the size of the dataset. The optimizer we chose is Adam [48]. The learning rate was set to $1\times 10^{-4}$.

Since the discovery of entanglement, researchers have explored various entanglement states, among which GHZ states and W states hold prominent positions in quantum mechanics [49]. These states exhibit non-classical correlation properties in multipartite quantum systems, having significance in fields like quantum communication and quantum teleportation [50,51,52,53]. Due to the pivotal roles of the GHZ states and the W states in quantum information processing, we consider generalized versions of GHZ states and W states as the target states for testing. The test states we choose are in the following form:

(18) $$|\psi_{p}\rangle =p|GHZ\rangle +\left(1-p\right)\sum_{i=1}^{n-1}|W_{i}\rangle$$

and

(19) $$|\phi_{p}\rangle =p\sum_{i=1}^{\lceil n/2\rceil -1}|W_{i}\rangle +\left(1-p\right)\sum_{\lfloor n/2\rfloor +1}^{n-1}|W_{i}\rangle ,$$

where

(20) $$|GHZ\rangle =\frac{|0\ldots 0\rangle +|1\ldots 1\rangle }{\sqrt{2}},$$

(21) $$|W_{i}\rangle =\frac{\sum^{C_{n}^{i}}\overset{\mathrm{There}\;\mathrm{are}\;i\;0}{\overset{︷}{|x_{1}\ldots x_{n}\rangle }}}{\sqrt{C_{n}^{i}}},$$

and p∈[0,1], with a step size of 0.005.

**Figure 2:**
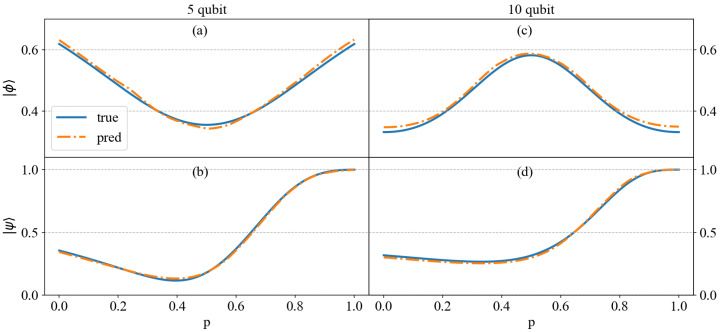
The trained neural network was used to fit the entanglement measure for the test states shown in Equations (18) and (19), for the 5-qubit and 10-qubit systems. The *x*-axis represents *p*, where p∈[0,1] takes 0.005 as the step. The *y*-axis for each figure represents the value of SE. The solid line represents the true values of SE calculated by Equation (9), and the dashed line represents the predicted values from the trained neural network. The upper two figures correspond to $|\phi_{p}\rangle$, while the lower section corresponds to $|\psi_{p}\rangle$. The corresponding MSE values are as follows: (**a**) $2.1797\times 10^{-5}$, (**b**) $5.8658\times 10^{-5}$, (**c**) $3.7323\times 10^{-5}$, and (**d**) $5.4019\times 10^{-5}$.

The performance of the trained neural network in predicting entanglement for five-qubit and ten-qubit systems is shown in Figure 2. It can be seen from the figure that the method proposed in this paper almost perfectly fits the true entanglement measures for both five-qubit and ten-qubit systems, demonstrating its efficiency and accuracy. For the five-qubit system, the error ranges for fitting |ϕ〉 and |ψ〉 are as follows: the maximum errors are $1.518\times 10^{-2}$ and $1.645\times 10^{-2}$, the minimum errors are $1.474\times 10^{-5}$ and $7.360\times 10^{-5}$, and the MSE are $8.6822\times 10^{-5}$ and $8.7521\times 10^{-5}$, respectively. Additionally, for the ten-qubit system, the maximum error for fitting |ϕ〉 is $1.792\times 10^{-2}$, the minimum error is $4.045\times 10^{-3}$, and the MSE is $1.2041\times 10^{-4}$; the maximum error for fitting |ψ〉 is $1.715\times 10^{-2}$, the minimum error is $3.160\times 10^{-5}$, and the MSE is $1.6268\times 10^{-4}$. It can be seen from these data that the model exhibits excellent performance in fitting the test states.

**Figure 3:**
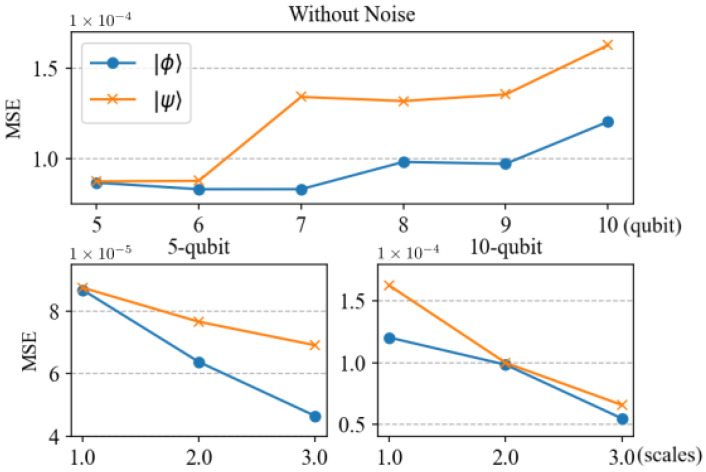
The MSE for predicting entanglement for the test states of |ϕ〉 (Equation (18)) and |ψ〉 (Equation (19)) using neural networks. The relationship between the number of qubits and MSE is shown in the upper figure, where the horizontal axis represents the number of qubits, ranging from 5 to 10. The relationship between different neural network scales and MSE is displayed in the lower two figures, with the horizontal values 1.0, 2.0, and 3.0 representing three scales of the neural networks. The one we previously used is 1.0, a fully connected neural network, consisting of six hidden layers with 100, 200, 400, 500, 200, and 100 neurons. Scales 2.0 and 3.0 correspond to networks where the number of neurons in each layer is multiplied by 2 or 3, respectively, compared to the 1.0 network. On the left side, systems with 5 qubits are displayed, while systems with 10 qubits are shown on the right side.

To further illustrate the effectiveness of the trained neural network, we also show the performance of the trained neural network in predicting entanglement for the test states shown in Equations (18) and (19) for the six-qubit to nine-qubit scenario. The results are shown in the upper figure of Figure 3. Similarly to the experiment for the five-qubit and ten-qubit scenario, *p* takes 0.05 as the step; thus, for each qubit number scenario, there are 200 data. We calculate the MSE for these test states and show them in Figure 3. It can be seen that the MSEs for these states are around $10^{-4}$; thus, the trained network fits these scenarios well. In addition, we note that with the increase in qubits from five to ten, the MSE shows a slight upward trend. This is because as the number of qubits increases, the dimension of the quantum state space grows exponentially, whereas the measurement method chosen in this paper only grows linearly. This mismatch in growth rates results in the loss of some information. However, it may be possible to improve this mismatch by increasing the number of neurons and the complexity of the model parameters. This is because larger models have more parameters, allowing them to more flexibly learn the complex features and patterns of the input data, thereby improving the model’s fitting ability. In addition, larger models typically better capture the non-linear relationships between data and have stronger generalization capabilities, performing better when faced with unseen data. In order to show whether prediction accuracy could be improved by increasing the size of the neural network model, we have introduced another two larger neural networks to predict the SE of the test states, and the results are shown in the lower figures of Figure 3. It can be seen that by increasing the size of the neural network model, the prediction accuracy continuously improves. Although increasing the size of the neural network model helps improve the accuracy of the prediction, the number of training parameters required is approximately 0.42 million, 1.68 million, and 3.78 million, respectively, which leads to increased training costs. However, the method proposed in this paper achieves high-precision predictions with fewer training parameters and within an acceptable error range. If further improvement in accuracy is required, increasing the size of the neural network model moderately may be considered. This approach can balance the relationship between accuracy and training costs while meeting the requirements of practical applications.

**Figure 4:**
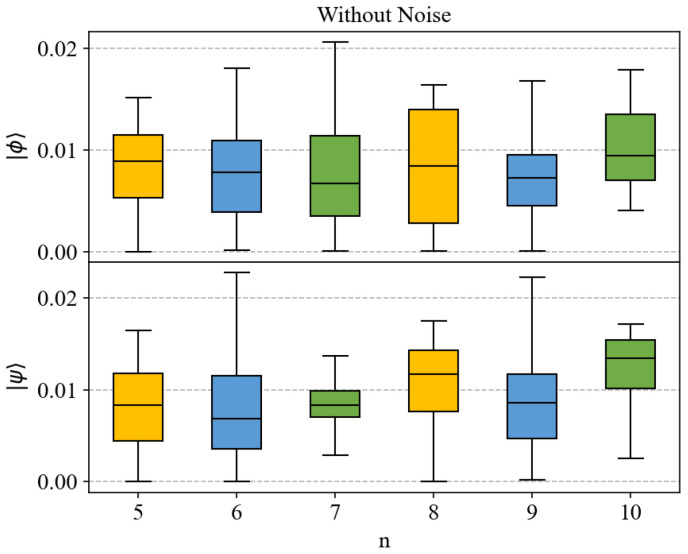
The boxplots of error distribution for predicting entanglement for the test states in Equations (18) and (19) using neural networks, across different numbers of qubits. The horizontal axis represents the number of qubits involved. The upper one is for the test state of Equation (18), and the lower one is for the test state of Equation (19). To differentiate, we labeled specific quantum states |ϕ〉 and |ψ〉 on the left side of the figures. The vertical axis represents MAE.

We also present boxplots of the errors that visualize the error distribution, including its central tendency and dispersion. Boxplots provide an intuitive way to observe data distribution, helping to understand the accuracy of model predictions. In the previous description, MSE was used as an error metric. However, MSE is not intuitive because it squares the errors, resulting in units that are inconsistent with the original data units. Therefore, Figure 4 shows the mean absolute error (MAE), representing the absolute difference between predicted and true values, to more intuitively present the distribution of errors. From Figure 4, we can compare the errors between different test states and numbers of qubits. It can be observed that although prediction accuracy may decrease with an increase in the number of qubits, most prediction errors are within the range of 0 to 0.02. This indicates that even when dealing with larger-scale quantum systems, neural networks can still predict entanglement measures relatively accurately.

The results above demonstrate the excellent performance of the method proposed in this paper for quantifying pure unknown multipartite entanglement states. With the continuous advancement of quantum technologies, larger-scale entanglement systems will be created in the future [54], and the method proposed in this paper provides a promising approach for studying these entanglement systems. This research opens up new possibilities for exploring more complex quantum entanglement phenomena, with the potential to drive advancements in the field of quantum information processing and play important roles in quantum computing, quantum communication, and other fields.

To further assess the performance of our model, we examine its ability to predict quantum random states. Specifically, we randomly generate 200 quantum states with qubit numbers ranging from n=5 to n=10, in the form of Equation (13), which represents a kind of Haar random state. For each case, we calculate the MSE between the actual and predicted values for the 200 states. The results, as shown in Table 1, indicate that the MSE is around $10^{-5}$ or $10^{-6}$, demonstrating the effectiveness of our trained model in predicting random states. This outcome is not unexpected, as the states used for network training follow the form of Equation (13), which involves interference with separable states based on the structure outlined in Equation (14).

## 4. Quantifying Multipartite Entanglement Pure State in Noise Case

In the description of the idealized scenarios, we considered a closed system. However, real-world quantum systems are rarely completely closed. Quantum systems typically undergo inevitable interactions with their surroundings, and these interactions manifest themselves as various forms of noise within the quantum system. This noise can arise from environmental factors, impurities, and imperfections in the quantum devices themselves [55]. Noise is also a major challenge in quantum technology. It can lead to information loss, causing severe impacts on applications that require high reliability, such as quantum computing and quantum communication. Noise may also induce irreversible losses in quantum states and interfere with qubits, limiting the performance of quantum systems. In the process of preparation of quantum states [56], noise often has an impact, leading to the manifestation of quantum states as mixed states. In practical applications of current quantum devices, focusing solely on entanglement quantification of pure states is no longer sufficient. Therefore, to more comprehensively adapt to the characteristics of modern quantum technology, we will further investigate the influence of the noise. We concentrate on depolarizing noise and random channel noise, two prevalent types of quantum noise. Depolarizing noise generally results from flaws in quantum gate operations, causing the qubit to become a random mixture of all possible states, effectively “depolarizing” it into a mixed state.

(22) $$\rho_{\mathrm{noise}}=\left(1-s\right)\rho +s\left(\frac{I}{2^{n}}\right),$$

where ρ represents the density matrix and *s* represents the probability.

Then, we consider a more general case, random channel noise, often described as bit-flip or phase-flip noise. Random channel noise can originate from multiple sources, including physical channel imperfections in qubit interactions or from external factors like electromagnetic interference. In random channel noise, each party may randomly generate bit flipand phase flip. Bit-flip noise introduces probabilistic changes in the bit values, potentially causing alterations in qubits that were originally in an entanglement state, thus disrupting their entanglement. On the other hand, phase-flip noise introduces randomness in the phase of quantum states, which may disturb specific phase relationships and consequently affect the maintenance and transmission of entangled states. Formally, they can be defined as [55]

(23) $$\left[\Lambda \left(\vec{s}\right)\right]\left(\rho \right)=\left(1-\sum_{j=1}^{2}s_{j}\right)\rho +\sum_{j=1}^{2}s_{j}U_{j}\rho U_{j}^{†}$$

for each party, where $U_{j}\in \left\{X,Z\right\}$, and $\vec{s}=\left\{s_{1},s_{2}\right\}$ represents the probability of the corresponding noise events. We use Equation (23) to apply channel noise to each qubit. After transmission in this quantum noise channel, the quantum state becomes the following:

(24) $$\left[\Lambda^{1}\left(\vec{s}\right)\right]⊗\left[\Lambda^{2}\left(\vec{s}\right)\right]⊗\ldots ⊗\left[\Lambda^{n}\left(\vec{s}\right)\right]\left(\rho \right).$$

In the case of random channel noise, this section will also employ the form after the action of Equation (24) as the decomposition of mixed states.

**Figure 5:**
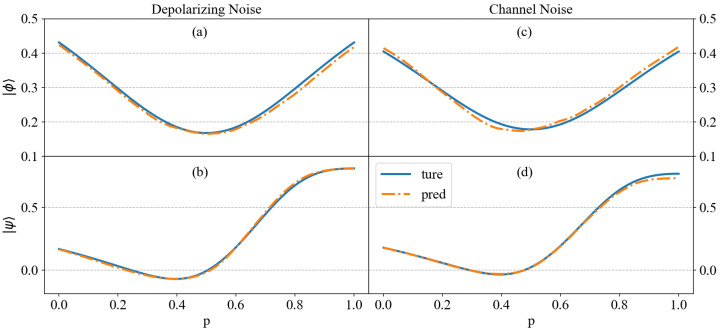
The trained neural network is used to fit the entanglement measure for 5-qubit test states |ϕ〉 and |ψ〉, under depolarizing noise and random channel noise. The horizontal axis represents the parameter *p* for |ϕ〉 and |ψ〉, where p∈[0,1], with a step size of 0.005. The corresponding MSE values are as follows: (**a**) $8.5237\times 10^{-5}$, (**b**) $9.7738\times 10^{-5}$, (**c**) $1.0371\times 10^{-4}$, and (**d**) $1.7841\times 10^{-4}$.

Figure 5 shows the results of data fitting for noise entanglement systems, with a specific focus on a five-qubit entanglement system, where SE is used to measure the entanglement of the noisy state. However, as mentioned in Section 2, quantifying entanglement in mixed states involves examining all potential decompositions of the state to determine the minimum value of Equation (5), which is impractical. With the entropy inequality, Equation (10) provides a lower bound for the C-SE across all possible state decompositions. We use the noise decomposition given in the paper as an approximation of the entanglement (Equation (24) for states under channel noise, and computational bases for *I* for depolarizing noise); the actual value may be higher than our approximation. However, because Equation (10) establishes a lower bound, the value derived from the given decomposition can also be lower than the true entanglement value. In our numerical experiments, the probability of depolarizing noise is set to 0.1. This moderate noise is often used to simulate a typical experimental environment where quantum gates and qubits are imperfect but not completely noisy. The noise level is realistic for near-term quantum devices in the Noisy Intermediate-Scale Quantum (NISQ) era, where noise is present but the quantum algorithms can still be executed with some error correction or mitigation techniques. Conversely, for channel noise, due to the accumulation of noise in each qubit system, the overall noise becomes significant, which increases the likelihood of a large deviation from the original state. Therefore, to ensure the efficiency of prediction, the probability of channel noise could not be set too high. Here, we choose the small value 0.02 as an example. For depolarizing noise, the error ranges for fitting test states |ϕ〉 and |ψ〉 are as follows: the maximum errors are $1.623\times 10^{-2}$ and $2.025\times 10^{-2}$, the minimum errors are $6.790\times 10^{-4}$ and $3.152\times 10^{-5}$, and the MSE are $8.5237\times 10^{-5}$ and $9.7738\times 10^{-5}$. Furthermore, for channel noise, the maximum error for fitting |ϕ〉 is $1.732\times 10^{-2}$, the minimum error is $5.671\times 10^{-5}$, and the MSE is $1.0371\times 10^{-4}$; the maximum error for fitting |ψ〉 is $3.616\times 10^{-2}$, the minimum error is $6.710\times 10^{-6}$, and the MSE is $1.7841\times 10^{-4}$. From this, it can be seen that the predictive performance is also excellent. Furthermore, from Figure 5, it can also be observed that negative values appear in the SE calculation process for the subset of |ψ〉 in the five-qubit system, leading to the appearance of negative values in the predicted results. This is because, in the study of mixed states, the calculated SE is not an exact value, but an estimate. When negative values occur, the method proposed in this paper may not accurately identify entanglement, which is a limitation of our approach. However, it still provides an approximation of the entanglement for the quantum states with noise.

**Figure 6:**
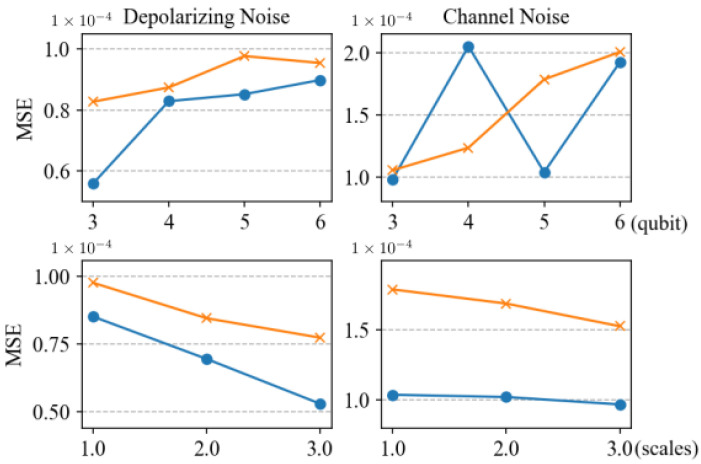
The MSE for predicting entanglement for the test states of Equations (18) and (19) under depolarizing noise Equation (22) and random channel noise Equation (23) using neural networks. The relationship between the number of qubits and MSE is shown in the upper two figures, where the qubit number is from 3 to 6. The left one is for depolarizing noise, and the right is for the channel noise, where the blue curves are for the test states of Equation (18), and the yellow ones are for the test state of Equation (19) under corresponding noise. The relationship between different neural network scales and MSE is displayed in the lower figures, with three different scales of the neural networks corresponding to the no-noise scenario described in Section 3.

**Figure 7:**
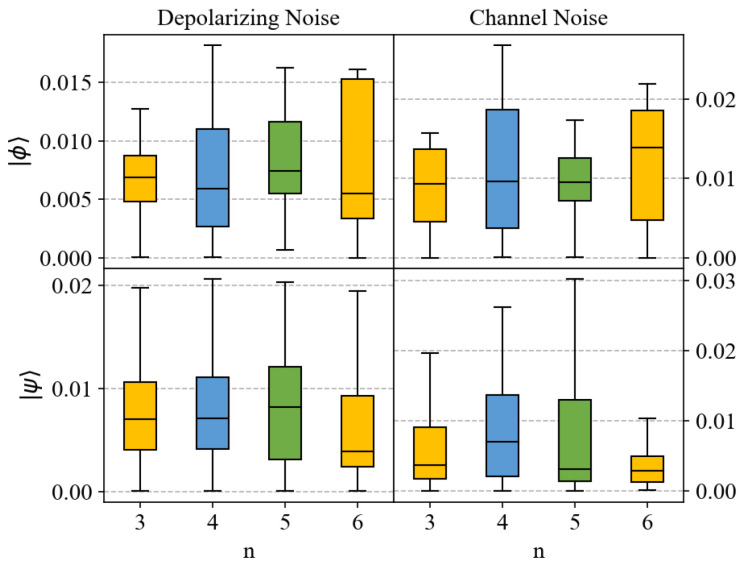
Box plots of error distribution for predicting entanglement for the test states of Equations (18) and (19) under depolarizing noise Equation (22) and random channel noise Equation (23) using neural networks, across different numbers of qubits with noise. The horizontal axis represents the number of qubits involved. The vertical axis represents the value of MAE. The top plot corresponds to the test state of Equation (18), and the bottom one corresponds to the test state of Equation (19).

Figure 6 illustrates the MSE for predictions from three-qubit systems to six-qubit systems, and the corresponding boxplots of prediction errors are presented in Figure 7. The phenomena observed in these figures and the reasons behind them are similar to those in the case without noise. In the presence of noise, the method proposed in this paper demonstrates excellent robustness. Compared to predicting pure states, the accuracy of predicting mixed states is slightly reduced. This phenomenon arises because mixed states possess more complex entanglement structures relative to pure states, and computing SE in the case of mixed states is more complicated. When calculating SE for mixed states, the last term in Equation (10) needs to be considered, which involves information about the entire system, increasing the difficulty of prediction. The method proposed in this paper relies mainly on local measurement results as feature inputs, without collective measurements, leading to some information loss. However, despite these challenges, our method still demonstrates satisfactory performance in handling mixed-state tasks. Furthermore, it can be observed that, compared to channel noise, the prediction performance is better for depolarizing noise. This is due to noise build-up in each qubit system, resulting in a low probability of state preservation. The computation of the entanglement entropy (SE) for mixed states requires traversing the entire decomposition space, which amplifies the error between estimated and true values under noise, thereby reducing predictive efficacy.

Furthermore, it can be observed from the top-right subplot in Figure 6 that under channel noise, the prediction error for the four-qubit entanglement system is relatively large. This is because for the four-qubit test state |ϕ〉, the expectation under the Pauli-X basis measurement is 0, while it is not 0 under other conditions. This results in multiple test states having the same features, increasing the difficulty of neural network prediction. Additionally, performing predictions under channel noise is inherently challenging because the introduction of noise makes the system’s behavior more complex and uncertain.

The observations above indicate that the method proposed in this paper can be applied to quantum states with noise, providing a promising approach for handling mixed states.

## 5. Comparison to the Current Methods

Our paper’s main contribution is quantifying unknown multipartite entanglement, using SE, derived from the local measurements on quantum states. Through noise decomposition for mixed states, the SE can be used as an entanglement approximation, indicating that true values might exceed this estimate. We have quantified the mixed entanglement for a 10-qubit system for the first time, with theoretical scalability to larger configurations, although our experiments were limited to 10 qubits on a laptop. Table 2 contrasts our method with established approaches. Unlike most existing techniques, including Roik et al.’s work which uses a set number of measurements for specific systems, our method relies on local measurements, leading to a linear growth in required measurements, in contrast to the quadratic or exponential increases seen with collective or tomographic approaches. This linear growth reduces the burden of classical communication and substantially reduces the need for measurement. Our approach, using SE for entanglement evaluation, is tailored for large systems. It offers a computational form adjusted for noise, which improves the application. Our method also examines more qubits than other techniques and incorporates noise considerations in qubit systems. Employing evaluation metrics like MAE (mean absolute error) and MA (mean error), we show reduced prediction errors and notable stability across different qubit counts and noise conditions.

## 6. Conclusions

Quantifying the entanglement of unknown quantum states is a challenging but significant task in the field of quantum information. In this paper, we harness the powerful capabilities of neural networks to tackle the complex task of quantifying the entanglement of unknown quantum states, achieving convincing and promising results. We first introduced the measurement settings for calculating the data label, specifically the squashed measurement. Subsequently, we verified the efficiency and accuracy of this method by showing the entanglement prediction results for different qubit systems. Moreover, as the size of the neural network model increases, the prediction accuracy consistently improves. However, high-precision predictions can still be obtained even with smaller-scale neural network models. Moreover, the research presented here demonstrates the potent noise resistance capability of the proposed method through the prediction of data from noise entanglement systems. Although some challenges arise when dealing with quantum states with noise, such as predicting negative values and a slight decrease in prediction accuracy, the method still exhibits excellent robustness and performance. In particular, when faced with depolarizing noise, the prediction results are more reliable, providing a promising approach for handling the noise present in actual quantum systems. This study also demonstrates the potential for scalability to large-scale quantum entanglement systems.

Compared to the approach proposed by Ma [31], our method is not limited to classification but can perform quantification, allowing more precise prediction and measurement of entanglement. Compared to other methods [33,34,40,41], our chosen measurement settings exhibit polynomial growth rather than exponential growth, making it efficient in handling larger-scale systems. In the future, efforts will be made to further theoretically derive a universal form of entanglement measure and perform quantification. Future work will also attempt to explore the theoretical role of collective measurements in entanglement measures. These efforts will improve the accuracy and generalizability of entanglement prediction and expand the applicability and practical use cases of the neural network.

## Figures and Tables

**Table 1:** MSE for predicting the entanglement of quantum random states. For each *n*, 200 quantum random states are generated randomly.

N	The True SE Interval	MSE
5	[0.7968, 0.9890]	7.4358 × $10^{-5}$
6	[0.8988, 0.9944]	3.9408 × $10^{-5}$
7	[0.9464, 0.9954]	2.4218 × $10^{-5}$
8	[0.9769, 0.9982]	1.8606 × $10^{-5}$
9	[0.9906, 0.9990]	9.9722 × $10^{-6}$
10	[0.9949, 0.9995]	6.8776 × $10^{-6}$

**Table 2:** Comparing neural network approaches for quantifying unknown entanglement.

Methods	Settings	Feature	Label	Systems	Noise	Precision
Lin [33]	Collective Measurement	$n^{2}$	Coherent Information	Two-qubit $n\in \left[3,10\right]$	Y	$10^{-3}$∼$10^{-2}$ (MSE)
Lin [33]	Collective Measurement	$2^{n}$	GME	Three-qubit	N	$10^{-5}$ (MSE)
Lin [33]	Collective Measurement	$n^{2}$	Relative Entropy of Entanglement	Two-qubit $n\in \left[2,4\right]$	Y	$10^{-5}$∼$10^{-3}$ (MSE)
Roik [40]	Collective Measurement	5∼10	Negativity	Two-qubit	N	10.02∼0.08 (MA)
Koutny [41]	Incomplete Measurement	4∼$\frac{6^{n}}{4}$	Concurrence	Two-qubit	Y	$10^{-5}$∼$10^{-1}$ (MAE)
Koutny [41]	Incomplete Measurement	4∼$\frac{6^{n}}{4}$	Mutual Information	*n*-qubit $n\in \left[2,5\right]$	N	$10^{-3}$∼$10^{-1}$ (MAE)
Ours	Local Measurement	3n	SE	*n*-qubit $n\in \left[3,10\right]$	N	$10^{-5}$∼$10^{-4}$ (MSE)

## Data Availability

The data involved in this article have been presented in the article.
